# EGFR-TKIs治疗失败的肺腺癌子宫内膜转移1例并文献复习

**DOI:** 10.3779/j.issn.1009-3419.2025.102.27

**Published:** 2025-07-20

**Authors:** Fangqian SHEN, Zuling HU, Hua YANG, Puyu LIU, Yuju BAI, Jianguo ZHOU, Hu MA

**Affiliations:** ^1^563000 遵义，遵义医科大学第二附属医院胸部肿瘤科; ^1^Department of Thoracic Oncology, The Second Affiliated Hospital of Zunyi Medical University, Zunyi 563000, China; ^2^563000 遵义，遵义医科大学第二附属医院腹部肿瘤科; ^2^Department of Abdominal Oncology, The Second Affiliated Hospital of Zunyi Medical University, Zunyi 563000, China; ^3^563000 遵义，遵义医科大学第二附属医院病理科; ^3^Department of Pathology, The Second Affiliated Hospital of Zunyi Medical University, Zunyi 563000, China

**Keywords:** 肺肿瘤, 子宫内膜肿瘤, 肿瘤转移, 免疫组化, EGFR-TKIs, Lung neoplasms, Endometrial neoplasm, Tumor metastasis, Immunohistochemistry, EGFR-TKIs

## Abstract

肺癌发病率、死亡率居高不下，是癌症相关死亡的首要原因。女性肺癌病理类型大多为肺腺癌，常见基因突变为表皮生长因子受体（epidermal growth factor receptor, *EGFR*）突变，EGFR-酪氨酸激酶抑制剂（EGFR-tyrosine kinase inhibitors, EGFR-TKIs）能有效改善患者预后。在原发性肺癌中，子宫内膜转移十分罕见，且很容易误诊为原发性生殖系统肿瘤，其出现提示患者预后不良。本文报道了1例*EGFR*突变阳性的晚期肺腺癌患者经EGFR-TKIs治疗失败后出现异常阴道流血，经活检证实子宫内膜转移，并对类似病例进行回顾性总结。

## Abstract (en)

The incidence and mortality rates of lung cancer remain high, making it the leading cause of cancer-related deaths. In women, the predominant histological subtype is lung adenocarcinoma, commonly associated with epidermal growth factor receptor (*EGFR*) mutations, and EGFR-tyrosine kinase inhibitors (EGFR-TKIs) can significantly improve patient prognosis. Metastasis of primary lung cancer to the endometrium is extremely rare and is often misdiagnosed as a primary reproductive system tumor, and its occurrence indicates poor prognosis. This article reports a case of an advanced lung adenocarcinoma patient with *EGFR* mutation, who developed abnormal vaginal bleeding after EGFR-TKIs treatment failure, and biopsy confirmed endometrial metastasis. A review of similar cases is also presented.

肺癌是中国发病率和死亡率最高的恶性肿瘤^[1]^，近年来，女性肺癌发病率逐年增高，多为肺腺癌，其中常见的基因突变之一是表皮生长因子受体（epidermal growth factor receptor, *EGFR*）的突变。EGFR-酪氨酸激酶抑制剂（EGFR-tyrosine kinase inhibitors, EGFR-TKIs）通过阻断EGFR蛋白的酪氨酸激酶活性，抑制肿瘤的生长，从而改善*EGFR*突变型肺腺癌患者的预后^[2]^。大多数患者在首诊时就已出现转移，最常见的转移部位为区域淋巴结、肺、肾上腺、骨骼和脑，而女性生殖系统转移相对少见，尤其是子宫内膜受累极为罕见。本研究报道了1例*EGFR*突变阳性的晚期肺腺癌患者经EGFR-TKIs治疗失败后出现子宫内膜转移的病例，并结合文献探讨了肺癌子宫内膜转移的可能机制及后续治疗，旨在为肺癌子宫转移患者的临床诊疗提供依据。

## 1 病例资料

患者女，38岁，既往史无特殊，无吸烟史，既往月经规律，经期5-7 d，周期29-30 d，月经量正常，偶有痛经，末次月经不详。2023年12月患者就诊于山东省公共卫生临床中心行肺部穿刺活检后诊断肺腺癌，随后就诊于北京大学肿瘤医院，胸部计算机断层扫描（computed tomography, CT）提示：左肺下叶背段结节，符合周围型肺癌表现：纵隔、双肺门及左侧锁骨上区多发淋巴结倾向转移；胸骨、腰椎多发骨质破坏，考虑转移。完善肿瘤基线评估及基因检测后临床诊断为：左肺下叶腺癌，cT3N3M1c，IVB期[锁骨上淋巴结、右侧肩胛骨、颈椎、胸椎、腰椎多发转移，*EGFR *21 L858R突变，*TP53* K132Q突变，美国癌症联合委员会（American Joint Committe on Cancer, AJCC）第8版癌症分期指南]。患者确诊后规律口服阿美替尼片110 mg *qd*靶向治疗，期间疗效评价为疾病稳定。2024年7月患者因呕吐不适就诊于遵义医科大学第二附属医院。入院后查颅脑磁共振成像（magnetic resonance imaging, MRI）无异常，基于RELAY研究，予抗血管生成治疗（贝伐珠单抗1000 mg *q3w*）联合阿美替尼110 mg *qd*治疗2个周期。后患者因全身多发骨转移处疼痛加重，行骨转移病灶放射治疗，处方剂量：44 Gy/2 Gy/22 f（颈、胸、腰椎），45 Gy/3 Gy/15 f（右侧肩胛骨），因无法耐受未完成处方剂量，实际照射剂量为：36 Gy/2 Gy/18 f（颈、胸、腰椎），45 Gy/3 Gy/15 f（右侧肩胛骨）。

2025年1月患者遵医嘱返院行第3个周期抗血管生成治疗联合靶向治疗，入院时查血红蛋白70 g/L，追问病史后患者诉已不规则阴道流血2月余，呈间歇性，每次量不多，暗红色，无腹痛不适，未予重视。妇科检查见宫颈肥大伴糜烂，其余无异常。完善人乳头瘤病毒分型检测及宫颈黏液薄层细胞学检查未见异常。阴道彩超提示：宫腔积液，宫颈外口处实质回声减低、欠均匀。后行宫腔镜下诊刮术，术中见子宫内膜毛糙，厚薄不均，血凝块及杂乱内膜充满整个宫腔。术后病理回示：送检子宫内膜组织内查见恶性肿瘤，结合病史及免疫组化表型，考虑转移性肺腺癌。免疫组化提示: 甲状腺转录因子-1（thyroid transcription factor-1, TTF-1）阳性，细胞角蛋白7（cytokeratin 7, CK7）阳性，新天冬氨酸蛋白酶A（novel aspartic proteinase A, Napsin A）阳性，配对盒基因8（paired box gene 8, PAX8）阴性，雌激素受体（estrogen receptor, ER）阴性，孕激素受体（progesterone receptor, PR）阴性（图1）。同时患者出现活动后劳累气促，超声提示大量胸腔积液，行胸腔闭式引流术，胸水送检结果提示恶性胸腔积液，考虑胸膜转移，遂予顺铂50 mg行胸腔灌注治疗2次，治疗后患者劳累气促症状明显好转。因患者出现肺腺癌胸膜及子宫内膜转移，考虑疾病进展，本拟再次行基因检测明确TKIs耐药原因及转移灶基因特征，但由于经济原因患者拒绝行基因检测。对于疾病进展后续治疗，本拟让患者入组临床研究，但是患者条件不符合，故未入组。HARMONi研究、KL264-01研究以及ORIENT-31研究提示依沃西单抗联合化疗、芦康沙妥珠单抗、信迪利单抗+贝伐珠单抗+化疗能显著改善EGFR-TKIs耐药患者的无进展生存期（progression-free survival, PFS）。结合患者经济情况，最终选择了基于ORIENT-31研究^[3]^的四药治疗方案，具体为：培美曲塞800 mg+卡铂500 mg+贝伐珠单抗800 mg+信迪利单抗200 mg（*q3w*）（图2）。治疗1个周期后患者因肺部感染于呼吸科住院治疗，后因病情加重于2025年4月去世。

**图1:**
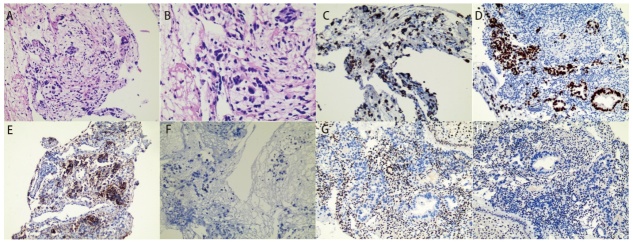
子宫内膜转移灶的组织病理学和免疫组织化学特征。A、B：（子宫内膜）转移性肺腺癌（HE染色，A：×100，B：×400）；C：CK7阳性（IHC染色，×200）；D：TTF-1阳性（IHC染色，×200）；E：Napsin-A阳性（IHC染色，×200）；F：PAX8阴性（IHC染色，×200）；G：ER阴性（IHC染色，×200）；H：PR阴性（IHC染色，×200）。

**图2:**
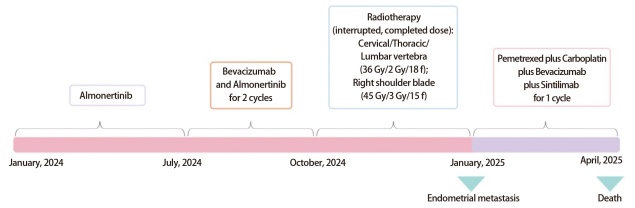
患者治疗时间线

**图3:**
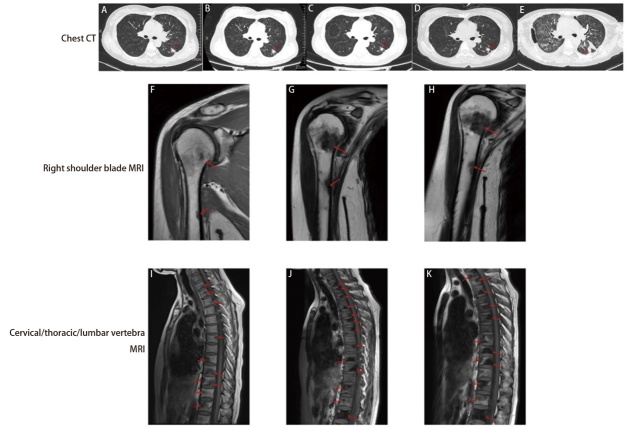
患者治疗过程中胸部原发肿瘤及骨转移灶变化。胸部CT扫描：A、B：口服阿美替尼片治疗期间（2024年5月25日、2024年7月11日）；C：阿美替尼联合贝伐珠单抗治疗2个周期后（2024年9月29日）；D：骨转移灶放疗期间（2024年11月20日）；E：培美曲塞+卡铂+贝伐珠单抗+信迪利单抗治疗后（2025年2月26日）。右肩关节MRI：F、G：口服阿美替尼治疗期间（2024年4月10日，2024年7月11日）；H：右肩关节转移灶放疗后（2024年12月10日）。颈/胸/腰椎MRI：I、J：口服阿美替尼治疗期间（2024年4月11日，2024年7月11日）；K：颈/胸/腰椎转移灶放疗后（2024年12月10日）。

## 2 讨论

肺癌是中国发病率最高的恶性肿瘤，也是癌症死亡的主要原因^[1]^，大约50%的患者在确诊时已发生转移。肺癌最常见的远处转移部位包括脑、骨骼、肝脏和肾上腺，而转移至生殖系统尤其子宫内膜极为罕见。有研究^[4]^认为，子宫内含有较多纤维和平滑肌组织，且子宫器官较小，减少了远端血液在其内流通，因此不利于恶性肿瘤播散。此外，子宫内膜的周期性脱离可能也限制了循环肿瘤细胞的定植。肺癌侵犯生殖系统并累及子宫内膜的情况极为少见。查阅国内外相关文献报道，仅有11例肺癌子宫内膜转移病例^[5-14]^（表1）。

**表1:** 肺腺癌子宫内膜转移既往病例报告

Author	Age (yr)	Histological type	Metastasis symptom	Histological finding in the lungs	Histological finding in the endometrial metastasis	EGFR mutation	Stage when first diagnosed	Treatment of lung cancer
Tiseo, et al.^[5]^	53	LUAD	Abnormal vaginal bleeding	CK7 and TTF-1 positive	CK7 and TTF-1 positive	Not described	IV	Chemotherapy
Hibi, et al.^[6]^	50	LUAD	Abnormal vaginal bleeding	Not described	Not described	Not described	Not described	Not described
Ahmad, et al.^[7]^	55	LUAD	None	CK7, TTF-1 and Napsin-A positive	CK7, TTF-1 and Vimentin positive, ER and PR negative	Not described	IIIB	Chemotherapy, radiotherapy and antiangiogenic therapy
	51	LUAD	Abnormal vaginal bleeding	CK7, Napsin-A, TTF-1 and MOC-31 positive	TTF-1 positive, ER and PR negative	Exon 21 p.L858R	IV	EGFR-TKIs
Kajimoto, et al.^[8]^	82	LUAD	Abnormal vaginal bleeding	CK7 and pankeratin positive, Napsin-A and TTF-1 negative	CK7 and pankeratin positive	Exon 21 p.L858R	IV	None
Cheng， et al.^[9]^	45	Lung solid mucinous cell adenocarcinoma	Abnormal vaginal bleeding	CK7 and TTF-1 positive	CK7 and TTF-1 positive, ER and PR negative	Not described	IV	Chemotherapy and radiotherapy
Anjali， et al.^[10]^	37	LUAD	None	CK7 and TTF-1 positive	TTF-1 positive	Exon 19 p.L858Arg, Exon 20 T790M	IV	Chemotherapy and EGFR-TKIs
Knox， et al.^[11]^	65	LUAD	Abnormal vaginal bleeding	Not described	CK7 and TTF-1 positive, CK20 and PAX8 negative	Not described	IV	Surgery
Bulutay et al.^[12]^	83	LUAD	Abnormal vaginal bleeding	Not described	TTF-1 and Napsin-A positive, PAX8 negative	Exon 19	IV	EGFR-TKIs
Chen， et al.^[13]^	50	LUAD	Abnormal vaginal bleeding	Not described	CK7, TTF-1 and Napsin-A positive, ER, PR and PAX8 negative	Not described	IV	None
Guo， et al.^[14]^	47	LUAD	Abnormal vaginal bleeding	Not described	CK7, TTF-1, Napsin-A and SPB positive	Exon 19	IV	Chemotherapy and EGFR-TKIs

LUAD: lung adenocarcinoma; EGFR: epidermal growth factor receptor; TKIs: tyrosine kinase inhibitors; CK20: cytokeratin 20; MOC-31: monoclonal antibody clone 31; SPB: surfactant protein B.

在这11例患者中，大多数初诊时即为IV期，伴有远处转移，本病例亦在首诊时即出现锁骨上淋巴结及全身多处骨转移。转移灶相关症状通常为阴道异常出血，这与原发性子宫内膜癌的首发症状相同。在明确子宫内膜肿瘤病灶来源时需借助免疫组化染色。90%的患者子宫内膜转移灶表现出TTF-1阳性，73%的患者CK7表达阳性，45%的患者Napsin-A表达阳性。TTF-1是甲状腺和肺上皮发育过程中产生的组织特异性转录因子，主要分布在间脑局部、甲状腺滤泡上皮和呼吸道上皮，是目前最常用的诊断肺腺癌的标志物。Napsin-A是一种显著表达于肺和肾脏的天门冬氨酸蛋白酶，在肺腺癌的诊断中具有较高的灵敏度和特异性。值得注意的是，女性生殖系统原发肿瘤如中肾样腺癌亦可表现出TTF-1阳性，而Napsin-A阳性除肺腺癌外还见于子宫内膜癌中的透明细胞癌类型。CK7阳性表达广泛见于乳腺、肺、胃、子宫等多个部位的肿瘤。因此，在鉴别肿瘤来源时，应同时结合多个部位的原发肿瘤相关特异性标志物进行判断。本文报告的患者子宫内膜组织CK7表达阳性，结合TTF-1、Napsin-A阳性，而PAX8、PR、ER等妇科相关肿瘤标志物表达阴性，最终考虑患者子宫内膜病灶为原发肺腺癌转移。

值得注意的是，在Kajimoto等^[8]^报告的病例中，患者肺及子宫内膜样本中均只有CK7和pankeratin表达阳性，而TTF-1、Napsin-A、PAX8、ER、PR等特异性标志物均呈阴性表达。仅凭免疫组化无法区分肿瘤是伴有子宫转移的原发性肺癌、伴有肺转移的原发性子宫癌还是起源于肺和子宫的双原发性癌。随后对肺和子宫内膜样本进行*EGFR*基因突变分析，两个样本中都鉴定出*EGFR*基因外显子21突变（L858R），而L858R突变在子宫内膜癌中尚未出现，这些结果强烈表明子宫肿瘤是肺腺癌的转移。因此，当肺和女性生殖道肿瘤同时存在时，免疫组化和基因突变检测有助于定位初始肿瘤部位。本例患者拒绝再次行基因检测，所以未对子宫内膜处病灶进行基因突变检测。

本文发现发生子宫内膜转移的肺癌患者病理学类型均为腺癌（表1），据报道，肺腺癌远处转移的发生率显著高于肺鳞状细胞癌^[15]^。这可能与肺腺癌中*EGFR*突变更常见有关，*EGFR*突变可能通过增强肿瘤细胞侵袭能力从而促进肿瘤转移。有研究^[16]^表明，EGFR通路和ER通路存在交互作用，雌激素和ER结合后能激活EGFR通路，通过上皮间充质转化促进肺癌细胞转移。此外，雌激素可上调子宫内膜中趋化因子配体12（C-X-C motif chemokine ligand 12, CXCL12）的表达^[17]^，CXCL12与趋化因子受体4（CXC chemokine receptor 4, CXCR4）结合后启动CXCR4/CXCL12轴，而*EGFR*突变通过激活CXCR4/CXCL12轴驱动肺腺癌转移。这些表明*EGFR*突变阳性的肺癌患者发生子宫内膜转移可能与某些特定因素如雌激素有关，但需要更多研究进一步验证。

本例患者出现阴道出血后并未重视，直至出现中度贫血后追问病史才得知此症状，若能尽早发现转移病灶或许能通过及时的干预改善患者的预后。鉴于肺腺癌转移至子宫内膜极为罕见，临床医生可能会忽略这种情况的发生，因此诊疗过程中若发现女性肺癌患者新发异常阴道出血、盆腔占位等相关临床症状时，需考虑肺癌子宫转移的可能，及时通过病理组织学和免疫组化检查明确诊断。此外，本文发现有2例发生子宫内膜转移的患者尚无异常阴道出血等相关表现（表1），而是在随诊检查中通过正电子发射断层扫描/CT（positron emission tomography/CT, PET/CT）意外发现子宫内膜转移灶。这提示在肺癌患者诊疗过程中，PET/CT对于发现子宫内膜转移具有重要意义。

本例患者经一线EGFR-TKIs治疗后失败，我们建议患者再次行基因检测明确EGFR-TKIs耐药机制，但患者拒绝。除可能发生的TKIs耐药突变外，*TP53*错义突变（K123Q突变）或许也是患者TKIs治疗失败的原因。Li等^[18]^研究表明，*TP53*突变与*EGFR*突变肺腺癌患者使用EGFR-TKIs疗效不佳相关，其中，发生*TP53*错义突变的患者PFS更短。

对于子宫转移灶的后续治疗，目前尚存在争议。对于远处转移部位仅限于女性生殖系统的患者，有研究^[19]^表明，外科姑息子宫切除术和双侧卵巢输卵管切除术可以明显减轻恶性肿瘤子宫转移患者的痛苦，并延长其生存期。在肺腺癌患者中，肺部原发病灶及子宫内膜转移灶的*EGFR*基因突变检测可能对后续治疗有重要指导意义。同时也有研究^[20]^认为，子宫转移是原发肿瘤终末期的表现，大多数患者同时还存在肿瘤广泛扩散于其他部位，因此不适合手术。对这类患者，基于原发肿瘤的全身系统治疗可能是他们的主要选择。本例患者初诊时即伴有全身多发骨转移，一线使用EGFR-TKIs治疗后失败。ORIENT-31研究^[4]^表明，免疫联合抗血管生成治疗及化疗可显著改善EGFR-TKIs耐药非小细胞肺癌患者的PFS。因此，在患者发生子宫内膜转移后，使用了培美曲塞+卡铂+贝伐珠单抗+信迪利单抗的四药治疗方案。遗憾的是，在完成第1个周期治疗后不久，患者因肺部感染加重去世，未能对TKIs耐药后的四药治疗方案进行疗效评价。考虑到患者在行子宫内膜活检明确转移时已出现2月余阴道流血，肿瘤向子宫内膜转移可能在更早前已发生，若能警惕异常症状并及时进行相应检查，或许能改善患者预后。
